# Association between Neonatal Intakes and Hyperglycemia, and Left Heart and Aortic Dimensions at 6.5 Years of Age in Children Born Extremely Preterm

**DOI:** 10.3390/jcm10122554

**Published:** 2021-06-09

**Authors:** Jawwad Hamayun, Lilly-Ann Mohlkert, Elisabeth Stoltz Sjöström, Magnus Domellöf, Mikael Norman, Itay Zamir

**Affiliations:** 1 Division of Pediatrics, Department of Clinical Science, Intervention and Technology, Karolinska Institutet, 141 52 Stockholm, Sweden; lilly-ann.mohlkert@sll.se (L.-A.M.); mikael.norman@ki.se (M.N.); 2 Department of Pediatric Cardiology, Sachs’ Children and Youth Hospital, Södersjukhuset, 118 83 Stockholm, Sweden; 3 Department of Food, Nutrition and Culinary Science, Umeå University, 901 87 Umeå, Sweden; elisabeth.stoltz.sjostrom@umu.se; 4 Department of Clinical Sciences, Pediatrics, Umeå University, 901 87 Umeå, Sweden; magnus.domellof@umu.se (M.D.); itay.zamir@umu.se (I.Z.); 5 Department of Neonatal Medicine, Karolinska University Hospital, 141 86 Stockholm, Sweden

**Keywords:** preterm birth, neonatal nutrition, hyperglycemia, echocardiography, left atrium, left ventricle, wall thickness, aorta

## Abstract

Survivors of extremely preterm birth (gestational age < 27 weeks) have been reported to exhibit an altered cardiovascular phenotype in childhood. The mechanisms are unknown. We investigated associations between postnatal nutritional intakes and hyperglycemia, and left heart and aortic dimensions in children born extremely preterm. Postnatal nutritional data and echocardiographic dimensions at 6.5 years of age were extracted from a sub-cohort of the Extremely Preterm Infants in Sweden Study (EXPRESS; children born extremely preterm between 2004–2007, *n* = 171, mean (SD) birth weight = 784 (165) grams). Associations between macronutrient intakes or number of days with hyperglycemia (blood glucose > 8 mmol/L) in the neonatal period (exposure) and left heart and aortic dimensions at follow-up (outcome) were investigated. Neonatal protein intake was not associated with the outcomes, whereas higher lipid intake was significantly associated with larger aortic root diameter (B = 0.040, *p* = 0.009). Higher neonatal carbohydrate intake was associated with smaller aorta annulus diameter (B = −0.016, *p* = 0.008). Longer exposure to neonatal hyperglycemia was associated with increased thickness of the left ventricular posterior wall (B = 0.004, *p* = 0.008) and interventricular septum (B = 0.004, *p* = 0.010). The findings in this study indicate that postnatal nutrition and hyperglycemia may play a role in some but not all long-lasting developmental adaptations of the cardiovascular system in children born extremely preterm.

## 1. Introduction

It has been estimated that preterm birth constitutes 10% of all live births, and that prematurity and its associated complications constitute the single most common cause of death worldwide in children below five years of age [1]. Advances in perinatal care have led to increased survival rates in high resource settings, especially among extremely preterm infants, where 4 out of 5 survive to 1-year-of-age [2]. Although this development has been very positive, concerns have been risen regarding the long-term health of preterm birth survivors. A unique cardiovascular phenotype characterized by smaller arteries and a more spherical heart shape has been observed in children and young adults born preterm [3,4,5,6]. These observations may provide insights as to why preterm birth has been associated with adult heart failure [7] and increased cardiovascular mortality in some [8] but not all epidemiologic studies [9].

The postnatal period for preterm infants is characterized by rapidly ongoing organ development that would normally happen in utero [10]. Preterm infants have considerably higher needs of nutrients and energy during the neonatal period compared to term-born infants and adequate nutrition is essential to ensure proper development and growth [11]. However, these nutritional needs are not easily met, and undernutrition as well as nutritional imbalances such as hyperglycemia have been reported to be common in preterm infants in neonatal intensive care [12,13]. Undernutrition and nutritional imbalances may have long term consequences. Both neonatal hyperglycemia and higher carbohydrate intake during the first eight postnatal weeks were associated with increased blood pressure at 6.5 years of age in children born extremely preterm [14]. Lewandowski et al. reported that breast-milk consumption in preterm-born neonates was associated with increased ventricular end-diastolic volume index and stroke volume index later in young adulthood [15]. Neonatal infusion of intravenous fat emulsion based on soybean oil (Intralipid™) and higher circulating cholesterol have been associated in a graded fashion with increased abdominal aortic stiffness in young adults born preterm [16], while maternal fish oil consumption during early pregnancy has been associated with reduced aortic stiffness in 9-year-old children born preterm [17].

There still remains an uncertainty regarding if and how specific nutritional elements provided early in life can affect long-term cardiovascular outcomes in preterm-born children. The aim of this study was to investigate associations between nutritional intakes and hyperglycemia during the neonatal period, and left heart and aortic dimensions in 6.5-year-old children born extremely preterm. The hypothesis of this study was that increased neonatal nutritional intakes, in particular protein intake, and exposure to hyperglycemia would be associated with increased left heart and aortic dimensions at 6.5 years of age.

## 2. Methods

### 2.1. Study Design and Participants

This study is a post-hoc retrospective cohort study using prospectively collected background and clinical data from the Extremely Preterm Infants in Sweden Study (EXPRESS), a population-based cohort including all 707 extremely preterm infants (<27 gestational weeks) born in Sweden between 1 April 2004 and 31 March 2007. Detailed descriptions of the EXPRESS cohort have been published previously [3,18,19].

**Figure 1:**
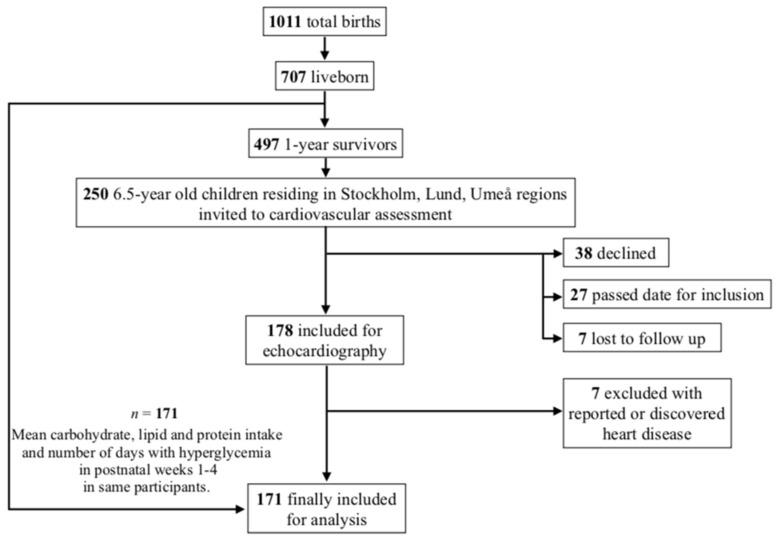
Study flow chart for participant selection included for analyses.

At 6.5 years of age ±3 months, children residing in three healthcare regions in Sweden (Stockholm, Lund, and Umeå), representing ~50% of survivors at this age, were invited to attend a pulmonary and cardiovascular follow-up visit. Out of 250 invited children, 38 children and their parents declined participation, 27 were excluded due to follow-up visit performed outside of the defined time frame, and seven were lost to follow up. Furthermore, seven children were excluded due to reported heart disease or heart disease discovered during echocardiography at follow-up. In total, 171 children were included in this study, as shown in Figure 1.

### 2.2. Exposure Variables

Daily intakes of protein, carbohydrates and lipids (g/kg/d), as well as energy (kcal/kg/d) during the neonatal period (first four postnatal weeks) were retrospectively obtained from hospital records. Both enteral (mostly maternal or donor breast milk, including human milk fortifiers) and parenteral nutrition intakes were registered and means of daily intakes were calculated. Nutritional data was registered and calculated using a computerized system (Nutrium Software by Nutrium AB, Umeå, Sweden). Number of days with hyperglycemia (defined as a blood glucose measurement > 8 mmol/L [20]) during the neonatal period was included as an exposure variable. A comprehensive description of data acquisition for nutrition [19] as well as blood glucose concentrations [13] has previously been published.

### 2.3. Outcome Variables

Follow-up assessment included echocardiography of the heart, aorta, and large arteries to determine dimensions and function. A comprehensive description of the cardiac assessments, including a comparison of preterm and term infants, has been published elsewhere [3]. Briefly, children born extremely preterm were found to exhibit a 3% to 5% smaller left ventricular length, width, and aortic valve annulus diameter, as well as a 5% lower left ventricular mass than matched control children born at term. Aortic dimensions included the end-systolic aortic annulus diameter, aortic root or sinus valsalva diameter, and end-diastolic abdominal aortic diameter. Left ventricle dimensions were assessed by diastolic left ventricle (LVd) length and width using an apical 4-chamber view. Left ventricle posterior wall (LV PW) thickness was assessed in end-diastole, and left ventricular (LV) mass was calculated using the Devereux formula [21]. Left ventricular sphericity was calculated as the ratio of left ventricle length to width. Left atrial (LA) measurements included end-systolic length and width in 4-apical chamber view as well as sphericity (calculated as the ratio of left atrial length to width). Interventricular septum thickness (IVS) was measured in end-diastole. Equations used to calculate stroke volume (SV), cardiac output (CO), relative wall thickness (RWT), aortic strain, and aortic stiffness have previously been described [3,4].

### 2.4. Statistical Analyses

Associations between individual neonatal macronutrient and energy intakes and number of days with hyperglycemia during the neonatal period, and left heart and aortic dimensions at 6.5 years of age were studied using multiple linear regression models. In case of an association between neonatal nutrition and cardiac outcome, regression analyses stratified on enteral or parenteral intakes in the neonatal period were also performed.

The following were considered as potential confounders of an association and were adjusted for in all models: gestational age, family history of cardiovascular disease (CVD; defined as myocardial infarction, coronary intervention, stroke, pharmaceutically treated hypertension or use of lipid-lowering drugs in first- or second-degree relatives), follow-up center (*n* = 40 in Lund, *n* = 83 in Stockholm, and *n* = 48 in Umeå), treated patent ductus arteriosus (PDA; defined as PDA requiring surgery or pharmaceutical treatment in the neonatal period), and days with neonatal mechanical ventilation treatment. Insulin treatment was regarded as a mediator and was therefore not adjusted for.

Body surface area (BSA) at 6.5 years of age was assumed to be associated with heart and aortic dimensions and all dimensional outcomes were therefore also adjusted for BSA (except for aortic strain and aortic stiffness index). BSA was calculated using Haycock’s formula [22] using registered measurements of height and weight.

Outcome variables that were not normally distributed were log-transformed prior to the statistical analysis. Regression coefficients (B) and their 95% confidence intervals (95% CI) were calculated to depict the change in outcome (the dependent variable) per one step increment in the exposure under study (the independent variable). *p*-values < 0.05 were regarded as indicators of statistically significant differences. Because of the potential for type 1 errors due to multiple comparisons, findings of this study should be interpreted as exploratory. All statistical analyses were performed using SPSS Statistical software (IBM Corp. Released 2015. IBM SPSS Statistics for Windows, Version 23.0. Armonk, NY, USA: IBM Corp).

### 2.5. Ethics

All parents and children received oral and written information, and the parents or legal guardians to participating children signed informed consent forms. The study was granted ethical permissions by the Regional Ethical Review Boards in Lund (no. 42/2004) and Stockholm (no. 520-31/2/2010, amendment no. 376-32/2011).

## 3. Results

### 3.1. Participant Characteristics

Participant characteristics are presented in Table 1. Mean gestational age at birth was 25.4 (SD ± 1.05) weeks and mean birth weight was 784 (SD ± 165) grams. Clinical characteristics of hyperglycemic infants included lower gestational age and more days with mechanical ventilation treatment [13].

### 3.2. Echocardiographic Outcomes

The echocardiographic outcome variables included at follow-up are shown in Table 2. The number of successful echocardiographic measurements varied for different outcome variables. The lowest number of successful measurements was 99 (57% of the cohort) for cardiac output and the highest number was 146 (85% of the cohort) for aorta annulus diameter.

### 3.3. Neonatal Nutrition, Hyperglycemia, and Left Atrial Dimensions

No significant associations were found between nutrition intakes during the neonatal period and LA dimensions at 6.5 years of age, as shown in Table 3.

An increasing number of days with neonatal hyperglycemia was significantly associated with increasing LA length and LA sphericity index. These associations remained significant when adjusted for carbohydrate intake (results not shown).

* Adjusted for family history of cardiovascular disease; gestational age; days with mechanical ventilation treatment and incidence of treated patent ductus arteriosus in the neonatal period; body surface area and center at follow-up.

### 3.4. Neonatal Nutrition, Hyperglycemia, and Left Ventricular Dimensions

Neonatal protein intake was not associated with LV dimensions at follow-up.

Carbohydrate intake during the neonatal period was significantly negatively associated with aorta annulus diameter at 6.5 years of age, as shown in Table 4. Stratified analyses by enteral and parenteral intakes revealed that the association between increasing neonatal carbohydrate intake and decreasing aorta annulus diameter was confined to parenteral intake (B = −0.006, *p* = 0.033) but not to enteral carbohydrate intake (B = 0.003, *p* = 0.347).

Increased lipid intake during the neonatal period was significantly associated with larger aortic sinus valsalva diameter. Analyzing enteral and parenteral intakes separately did not result in any significant associations between neonatal lipid intake and LV dimensions.

Higher energy intake during the neonatal period was significantly associated with shorter LVd Length. Separate analyses for enteral and parenteral neonatal energy intakes did not contribute to the findings.

An increasing number of days with hyperglycemia during the neonatal period was significantly associated with shorter LV length. This association remained significant when adjusted for carbohydrate intake but not when adjusted for energy intake (results not shown).

### 3.5. Neonatal Nutrition, Hyperglycemia, and Cardiac Functional Volumes

No significant associations were found between neonatal nutrition or days with hyperglycemia and functional cardiac volumes at 6.5 years of age, as shown in Table 5.

### 3.6. Neonatal Nutrition, Hyperglycemia and LV Wall Thickness

Neither neonatal protein nor lipid intakes were associated with LV wall thickness at follow-up, as shown in Table 6.

An increasing number of days with hyperglycemia during the neonatal period was significantly associated with thicker IVS and LV PW. These associations remained significant when adjusted for carbohydrate intake (results not shown).

### 3.7. Neonatal Nutrition, Hyperglycemia, and Abdominal Aortic Dimension and Stiffness

No significant associations were found between nutrition intakes or days with hyperglycemia during the neonatal period and abdominal aortic dimension or stiffness at 6.5 years of age, as shown in Table 7.

## 4. Discussion

In this retrospective cohort study, longer periods with hyperglycemia during the neonatal period were significantly associated with increased end-diastolic interventricular septum and left ventricle posterior wall thickness, left atrial length, and sphericity in 6.5 year-old children born extremely preterm. In addition, higher neonatal lipid intake was significantly associated with increased end-diastolic aortic sinus valsalva diameter, whereas a higher carbohydrate intake was significantly associated with a smaller aorta annulus diameter. Energy intakes were not significantly associated with these outcomes, suggesting that the above-mentioned findings are attributed to other mechanisms than energy intake.

Preterm birth has previously been associated with an altered cardiovascular morphology later in life [3,4,5,6,23]. The children studied herein were found to have smaller left ventricles and aortic valve annulus diameters as well as higher blood pressure at 6.5 years of age compared with children born at term [3,24]. The stroke volume and cardiac output were also lower in children born extremely preterm than in controls born at term. However, after adjusting for body surface, only stroke volume was significantly lower in children born extremely preterm than in controls born at term (adjusted mean difference: −0.8 mL/m^2^) [3].

The results of this study suggest that early-life nutrition and hyperglycemia may be associated with long-term morphology of the cardiovascular system in children born extremely preterm. Associations between maternal nutrition during pregnancy and the cardiovascular system in the offspring have previously been reported [25]; Obermann–Borst et al. reported that a maternal diet during early pregnancy rich in fish and seafood was associated with a decreased risk of congenital heart defects. On the other hand, reduced folate intake in pregnancy has been associated with not only congenital heart defects [26] but also with reduced neonatal endothelial function, which is linked to metabolic and sympathetic abnormalities and to coronary heart disease later in life [27]. 

In rat-models, maternal undernutrition has been associated with deficient development of the aorta and changes in collagen and elastin expression in the perinatal period, as well as reduced aortic wall thickness in offspring [28,29]. Protein deficiency in pregnant rats has also been associated with decreased heart weight in offspring [30]. These studies suggest that nutrition during the fetal period may have a role in the long-term development of the cardiovascular system. In contrast to our hypothesis, protein intake in extremely preterm infants was not found to be associated with left ventricular thickness.

Breastmilk consumption in low birthweight preterm infants has been associated with beneficial cardiac morphology and improved ventricular function in young adults as compared with formula feeding [15]. Lewandowski et al. [16] have also shown that neonatal intake of intravenous fat emulsion based on soybean oil (Intralipid™) and corresponding circulating cholesterol in preterm infants was associated with increased aortic stiffness in early adulthood. On the other hand, Bryant et al. [17] found that maternal fish oil consumption during pregnancy was associated with lower aortic stiffness at nine years of age in children born preterm. The exposures in these two studies differed a great deal, which might account for the different results reported. The exact mechanisms in which lipid intake in the postnatal period might affect aortic diameter in children born preterm is unknown, and no significant associations between macronutrient intakes and aortic stiffness were found in the current study. 

In a previous study of the EXPRESS cohort, increased intakes of carbohydrates and hyperglycemia during the neonatal period were significantly and independently associated with higher systolic and diastolic blood pressures at 6.5 years of age [14]. In the present study, total and parenteral carbohydrate intakes were linked to smaller aortic annulus of the LV outflow tract, and hyperglycemia in the neonatal period was associated with increased LV wall thickness at a 6-year follow-up. Fetal adaptations to hyperglycemia include hypertrophy of the interventricular septum [31] and shorter and narrower ventricles [32], and children to mothers with pregestational diabetes have been reported to exhibit left ventricular hypertrophy [33]. Hyperglycemia in preterm neonates has also been reported to be associated with cardiac septal hypertrophy and right ventricular dysfunction [11]. Although the extremely preterm born children in this study did not exhibit increased left ventricular mass on a group level [3], the longer-term association between neonatal hyperglycemia and LV mass remains to be established. The observation that neonatal hyperglycemia was associated not only to increased LV wall thickness but also to higher blood pressure [14] adds to the significance of glucose metabolism as a potentially important mechanistic pathway for metabolic programming in infants born extremely preterm.

While reduced length of the LV has been described as an early life adaptation to hyperglycemia [32], an explanation for an elongation of the left atrium associated with hyperglycemia is lacking. Previous studies of the diastolic filling pattern indicated a stiffer LV wall in children born extremely preterm than in control children born at term [3]. Whether or not such increased workload on the left atria contributes to its shaping and if hyperglycemia adds to such processes remains to be studied.

The strengths of this study include its prospective population-based cohort design, allowing good generalizability. Furthermore, comprehensive data regarding nutritional intakes and blood glucose concentrations could be matched with prospectively-registered echocardiographic data recorded at follow-up at 6.5 years of age. Breastmilk was analyzed for macronutrient components and energy, allowing for stratified analyses. The same echocardiography technician conducted the procedure at the follow-up visit at all three regional sites, thereby increasing intra-rater reliability and eliminating the possibility of inter-rater differences. The limitations of this study include the retrospective nature of nutrition and glucose data collection. The study was not powered to investigate the outcomes reported. Nutrition intakes in this cohort were lower than currently recommended intakes, which might affect the generalizability of the results to current practice [19]. Furthermore, the outcomes of LV mass, BSA, aortic strain, the aortic stiffness index, SV, and CO were calculated using equations using other echocardiography measurements, and these measurements were not available for all children. Causality cannot be inferred from the associations described in this study and remaining confounding factors cannot be ruled out. 

## 5. Conclusions

In children born extremely preterm, postnatal nutrition as well as neonatal hyperglycemia showed different degrees of associations with some cardiac dimensions at 6.5 years of age. Further studies are needed in order to investigate how early-life nutrition and hyperglycemia might affect the development of the cardiovascular system, and how these factors can be modified to improve the long-term health outcomes of people born preterm.

## Figures and Tables

**Table 1:** Characteristics of the study population.

Participant Characteristic, *n* = 171	Mean ± SD/Number (%)
**Maternal Characteristics**	
Age, years	31.5 ± 5.45
Family history of cardiovascular disease	123 (74)
**Neonatal Characteristics**	
Males	94 (55)
Birth weight, g	784 ± 165
Gestational age, weeks	25.4 ± 1.05
Treated patent ductus arteriosus	100 (59)
Mechanical ventilation treatment, days	10.8 ± 8.78
Mean protein intake, g/kg/d	2.6 ± 0.30
Mean carbohydrate intake, g/kg/d	11.2 ± 1.25
Mean lipid intake, g/kg/d	4.29 ± 1.04
Mean energy intake, kcal/kg/d	95.1 ± 11.8
Prevalence of at least 1 day with hyperglycemia (blood glucose > 8 mmol/L)	157 (91.8)
Days with hyperglycemia (blood glucose > 8 mmol/L)	6.84 ± 6.03 range: 0–27 days
Insulin treatment	15 (8.8)
**AT FOLLOW-UP (6.5 years)**	
Age, years	6.6 ± 0.19
Height, cm	118 ± 5.6
Weight, kg	20.6 ± 3.6
Body mass index, kg/m^2^	14.7 ± 1.6
Body surface area, m^2^	0.82 ± 0.09

**Table 2:** Left heart and aortic dimensions in 6.5-year-old children born extremely preterm.

Echocardiographic Outcome Variable	Mean	±SD	Children with Successful Measurements, *n* (%)	
**Left Atrial Dimensions**				
Left atrial length in A4C view systole, mm	36.5	±5.1	110	(64)
Left atrial width in A4C view systole, mm	27.3	±2.8	110	(64)
Left atrial sphericity index	1.34	±0.19	110	(64)
**Left Ventricular Dimensions**				
Left ventricle length in A4C view diastole, mm	54.9	±4.1	112	(65)
Left ventricle width in A4C view diastole, mm	34.7	±2.7	114	(66)
Left ventricle sphericity index	1.59	±0.16	112	(65)
Aorta annulus diameter systole, mm	13.9	±1.1	146	(85)
Aorta sinus valsalva diameter diastole, mm	18.9	±1.4	106	(62)
**Volumes**				
Stroke volume, mL	11.7	±2.8	109	(64)
Cardiac output, mL/min	1029	±299	99	(58)
**Wall Thickness**				
Interventricular septum diastole, mm	5.7	±0.8	113	(66)
Left ventricle posterior wall diastole, mm	5.4	±0.8	113	(66)
Relative wall thickness	0.32	±0.036	110	(64)
Left ventricle mass, g	48.2	±10.7	113	(66)
**Aorta**				
Aortic strain, %	28.5	±8.4	108	(63)
Aortic stiffness index	2.58	±4.99	108	(63)
Abdominal aorta diastole, mm	7.3	±0.8	108	(63)

A4C—Apical 4-chamber view.

**Table 3:** Associations between macronutrient and energy intakes and hyperglycemia during the neonatal period with left atrial dimensions at 6.5 years of age in children born extremely preterm.

Neonatal Nutrition and Hyperglycemia	Left Atrial (LA) Dimensions * B (95% CI), *p*-Value		
	LA Length Systole, mm	LA Width Systole, mm	LA Sphericity Index
Mean daily protein intake per 1 g/kg/d increase	0.126 (−0.188, 0.439), *p* = 0.428	0.034 (−0.150, 0.218), *p* = 0.715	0.024 (−0.107, 0.155), *p* = 0.718
Mean daily carbohydrate intake per 1 g/kg/d increase	0.050 (−0.021, 0.120), *p* = 0.168	0.013 (−0.029, 0.055), *p* = 0.529	0.010 (−0.020, 0.039), *p* = 0.527
Mean daily lipid intake per 1 g/kg/d increase	−0.018 (−0.116, 0.080), *p* = 0.715	0.033 (−0.024, 0.090), *p* = 0.256	−0.024 (−0.065, 0.016), *p* = 0.239
Mean daily energy intake per 1 kcal/kg/d increase	0.001 (−0.007, 0.010), *p* = 0.769	0.003 (−0.002, 0.008), *p* = 0.186	−0.001 (−0.005, 0.002), *p* = 0.430
N days with hyperglycemia > 8 mmol/L per 1 day increase	0.023 (0.003, 0.043), ***p*** **= 0.028**	−0.002 (−0.014, 0.10), *p* = 0.742	0.009 (0.001, 0.018), ***p*** **= 0.035**

* Adjusted for family history of cardiovascular disease; gestational age; days with mechanical ventilation treatment and incidence of treated patent ductus arteriosus in the neonatal period; body surface area and center at follow-up.

**Table 4:** Associations between macronutrient and energy intakes and hyperglycemia during the neonatal period with left ventricular dimensions at 6.5 years of age in children born extremely preterm.

Neonatal Nutrition and Hyperglycemia	Left Ventricular (LV) Dimensions * B (95% CI), *p*-Value				
	LVd Length, mm	LVd Width, mm	LV Sphericity Index	Aorta Annulus Diameter, mm	Aorta Sinus Valsalva Diameter, mm
Mean daily protein intake per 1 g/kg/d increase	−0.155 (−0.397, 0.086) *p* = 0.205	−0.059 (−0.225, 0.107) *p* = 0.483	−0.018 (−0.119, 0.084) *p* = 0.731	−0.034 (−0.087, 0.018) *p* = 0.200	0.075 (−0.027, 0.177) *p* = 0.147
Mean daily carbohydrate intake per 1 g/kg/d increase	−0.047 (−0.102, 0.008) *p* = 0.092	−0.013 (−0.051, 0.024) *p* = 0.483	−0.006 (−0.029, 0.017) *p* = 0.584	−0.016 (−0.028, −0.004) ***p* = 0.008**	−0.018 (−0.041, 0.004) *p* = 0.109
Mean daily lipid intake per 1 g/kg/d increase	−0.072 (−0.145, 0.01) *p* = 0.054	0.026 (−0.025, 0.077) *p* = 0.307	−0.032 (−0.062, −0.001) *p* = 0.042 **	0.008 (−0.008, 0.023) *p* = 0.338	0.040 (0.011, 0.070) ***p* = 0.009**
Mean daily energy intake per 1 kcal/kg/d increase	−0.009 (−0.015, −0.002) ***p*** **= 0.009**	0.001 (−0.004, 0.006) *p* = 0.654	−0.003 (−0.006, 0.000) *p* = 0.041 ***	−0.000 (−0.002, 0.001) *p* = 0.628	0.002 (−0.001, 0.005) *p* = 0.163
N days with hyperglycemia > 8 mmol/L per 1 day increase	−0.016 (−0.032, −0.001) ***p*** **= 0.041**	−0.001 (−0.012, 0.010) *p* = 0.835	−0.005 (−0.012, 0.002) *p* = 0.182	0.001 (−0.003, 0.004) *p* = 0.685	0.002 (−0.004, 0.009) *p* = 0.517

* Adjusted for family history of cardiovascular disease; gestational age; days with mechanical ventilation treatment and incidence of treated patent ductus arteriosus in the neonatal period; body surface area and center at follow-up. ** F-test for full model not significant (*p* = 0.432). *** F-test for full model not significant (*p* = 0.429). LVd Length = End diastolic left ventricular length. LVd Width = End diastolic left ventricular width.

**Table 5:** Associations between macronutrient and energy intakes and hyperglycemia during the neonatal period with cardiac functional volumes at 6.5 years of age in children born extremely preterm.

Neonatal Nutrition and Hyperglycemia	Cardiac Functional Volumes * B (95% CI), *p*-Value	
	SV, mL	CO, mL/min
Mean daily protein intake per 1 g/kg/d increase	1.191 (−0.677, 3.059) *p* = 0.209	149.328 (−66.983, 365.639) *p* = 0.173
Mean daily carbohydrate intake per 1 g/kg/d increase	−0.205 (−0.618, 0.209) *p* = 0.328	−12.333 (−59.823, 35.157) *p* = 0.607
Mean daily lipid intake per 1 g/kg/d increase	0.438 (−0.109, 0.984) *p* = 0.115	50.274 (−13.299, 113.848) *p* = 0.120
Mean daily energy intake per 1 kcal/kg/d increase	0.021 (−0.027, 0.070) *p* = 0.390	2.970 (−2.609, 8.550) *p* = 0.293
N days with hyperglycemia > 8 mmol/L per 1 day increase	−0.075 (−0.192, 0.043) *p* = 0.209	−13.421 (−27.156, 0.313) *p* = 0.055

* Adjusted for family history of cardiovascular disease; gestational age; days with mechanical ventilation treatment and incidence of treated patent ductus arteriosus in the neonatal period; body surface area and center at follow-up. CO = Cardiac Output. SV = Stroke Volume.

**Table 6:** Associations between macronutrient and energy intakes and hyperglycemia during the neonatal period with left ventricular wall thickness at 6.5 years of age in children born extremely preterm.

Neonatal Nutrition and Hyperglycemia	LV Wall Thickness (WT) * B (95% CI), *p*-Value			
	IVS Diastole, mm	LV PW Diastole, mm	Relative WT	LV Mass, g
Mean daily protein intake per 1 g/kg/d increase	0.009 (−0.044, 0.062) *p* = 0.746	0.001 (−0.051, 0.052) *p* = 0.979	0.006 (−0.021, 0.032) *p* = 0.670	1.839 (−3.776, 7.454), *p* = 0.517
Mean daily carbohydrate intake per 1 g/kg/d increase	−0.008 (−0.020, 0.004) *p* = 0.197	0.001 (−0.011, 0.012) *p* = 0.891	0.021 (−0.006, 0.006) *p* = 0.995	−0.481 (−1.756, 0.795), *p* = 0.456
Mean daily lipid intake per 1 g/kg/d increase	−0.003 (−0.019, 0.013) *p* = 0.736	0.011 (−0.005, 0.026) *p* = 0.169	0.001 (−0.007, 0.009) *p* = 0.773	0.843 (−0.842, 2.528), *p* = 0.323
Mean daily energy intake per 1 kcal/kg/d increase	−0.001 (−0.002, 0.001) *p* = 0.399	0.001 (0.000, 0.002) *p* = 0.202	0.000 (−0.001, 0.001) *p* = 0.731	0.043 (−0.105, 0.191), *p* = 0.564
N days with hyperglycemia > 8 mmol/L per 1 day increase	0.004 (0.001, 0.008) ***p* = 0.010**	0.004 (0.001, 0.008) ***p* = 0.008**	0.003 (0.001, 0.004) *p* = 0.002 **	0.331 (−0.027, 0.688), *p* = 0.069

* Adjusted for family history of cardiovascular disease; gestational age; days with mechanical ventilation treatment and incidence of treated patent ductus arteriosus in the neonatal period; body surface area and center at follow-up. ** F-test for full model not significant (*p* = 0.087). IVS diastole = End-diastolic interventricular Septum Thickness. LV = Left Ventricle. LV PW = Left Ventricular Posterior Wall Thickness.

**Table 7:** Associations between neonatal macronutrient and energy intakes and hyperglycemia during the neonatal period with abdominal aortic diameter and stiffness at 6.5 years of age in children born extremely preterm.

Neonatal Nutrition and Hyperglycemia	Abdominal Aortic Diameter * and Stiffness B (95% CI), *p*-Value		
	Abdominal Aorta End-Diastolic Diameter, mm	Aortic Strain, %	Aortic Stiffness Index (Log)
Mean daily protein intake per 1 g/kg/d increase	0.001 (−0.057, 0.059) *p* = 0.978	0.153 (−6.004, 6.310) *p* = 0.961	−0.110 (−0.261, 0.040) *p* = 0.150
Mean daily carbohydrate intake per 1 g/kg/d increase	−0.010 (−0.022, 0.003) *p* = 0.132	−0.010 (−1.345, 1.324) *p* = 0.988	−0.011 (−0.044, 0.022) *p* = 0.519
Mean daily lipid intake per 1 g/kg/d increase	0.014 (−0.003, 0.031) *p* = 0.110	−0.830 (−2.643, 0.983) *p* = 0.366	−0.006 (−0.051, 0.039) *p* = 0.804
Mean daily energy intake per 1 kcal/kg/d increase	0.000 (−0.001, 0.002) *p* = 0.614	−0.057 (−0.211, 0.098) *p* = 0.470	−0.001 (−0.005, 0.003) *p* = 0.535
N days with hyperglycemia > 8 mmol/L per 1 day increase	−0.002 (−0.006, 0.002) *p* = 0.261	0.002 (−0.393, 0.398) *p* = 0.990	−0.002 (−0.011, 0.008) *p* = 0.747

* Adjusted for family history of cardiovascular disease; gestational age; days with mechanical ventilation treatment and incidence of treated patent ductus arteriosus in the neonatal period; body surface area and center at follow-up.

## Data Availability

The Swedish Ethics Review Authority only granted publication of aggregated data, which means that individual data cannot be shared.
